# Functional Characterization and Toxicity of Pectin from Red Chilto Fruit Waste (Peels)

**DOI:** 10.3390/plants12142603

**Published:** 2023-07-10

**Authors:** María Eugenia Orqueda, Iris Catiana Zampini, Sebastian Torres, María Inés Isla

**Affiliations:** 1 Natural Products Research Laboratory (LIPRON), Institute of Bioprospecting and Plant Physiology (INBIOFIV-CONICET-UNT), Facultad de Ciencias Naturales e Instituto Miguel Lillo, Universidad Nacional de Tucumán, San Lorenzo, 1469, San Miguel de Tucumán T4000, Argentina; eorqueda@yahoo.com.ar (M.E.O.); zampini@csnat.unt.edu.ar (I.C.Z.); sebatk@hotmail.com (S.T.); 2 Biolates Network for Sustainable Use of Ibero-American Vegetable Biomass Resources in Cosmetics (Biolates CYTED), San Miguel de Tucumán T4000, Argentina; 3 Facultad de Ciencias Naturales e IML, Universidad Nacional de Tucumán, San Miguel de Tucumán T4000, Argentina

**Keywords:** red chito peel, pectin, by-products, functional properties, toxicity

## Abstract

Background: Food and agricultural wastes constitute a rich source of functional ingredients for the food, pharmaceutical, and cosmetic industries. In this context, by-products from the red variety of *Solanum betaceum* fruits (chilto) from Northwestern Argentina are suitable sources for pectin extraction. Methods: In this study, pectin from the peels of red chilto fruits was extracted and characterized. Results: The recovery yield of red chilto peel pectin was about 24%, and it was co-extracted with 40.0 mg phenolic compounds, 6.5 mg anthocyanins, and 4.7 g proteins per 100 g of pectin. The pectin obtained from red chilto showed proper technological functionality displaying water and oil holding capacities of 4.2 and 2.0%, respectively, an emulsifying capacity of 83%, emulsion stability of 87.5%, foaming capacity of 21.1%, and foaming stability of 79.1%. The pectin displayed antioxidant activity with the ability to scavenge ABTS radical, superoxide anion, and H_2_O_2_. The polysaccharide exhibited in vitro hypoglycemic potential and inhibited the α-amylase enzyme, retarded glucose diffusion, and improved the cellular uptake of glucose in a *Saccharomyces cerevisiae* model. The extract was non-toxic on acute toxicity tests. Conclusions: Red chilto pectin showed potential as a new and safe functional ingredient for the design of foods, health products, and cosmetics.

## 1. Introduction

Pectins are heterogeneous polysaccharides that constitute a part of the primary cell walls and middle lamellae of cells of higher plants [1]. Structurally, pectin is a heteropolysaccharide composed of α-(1–4)-linked D-galacturonic acid and other saccharides like galactose, arabinose, and rhamnose. Pectin can have varying degrees of esterification (methylation and/or acetylation) of the carboxylic acid residues. This polysaccharide is a high-value functional food ingredient frequently used as a gelling and stabilizing agent in jams and jellies [2] and could also be used in cosmetics. Pectin is commonly found in citrus, apples, pears, plums, or guavas, among other fruits. However, the substitution in food industries of commercial pectins for pectins extracted from other sources has increased drastically in recent years [3]. The expenditure for pectin production used in different industries is generally higher than 11 USD/kg, but various food wastes portray alternatives to obtaining pectin and could represent a reduction in the costs for production [4]. About 1.3 billion tonnes of food is wasted annually worldwide, representing about a third of total product for human consumption. Most of this waste consists of fruits and vegetables (50% of the waste generated) and occurs mainly during the processing and post-harvest stages [5]. Therefore, there is a growing need to recover value-added components such as pectin from fruit peels and pomace as a food waste valorization process that could offer economic and environmental benefits [6]. There are numerous reports of the extraction and characterization of pectins from fruit peels such as citrus, passion fruit, melon, and eggplant, among others [6,7,8,9].

*Solanum betaceum*, also known as “tree tomato”, “tamarillo”, or “chilto” in Argentina, is an edible native tree to the Andean region of South America that grows naturally in Argentina’s Yungas ecoregion. Three varieties of native chilto fruits are described in Northwestern Argentina: red, orange, and red/orange. The chilto fruits were mainly used for consumption by local communities for a long time in Argentina. Currently, the cultivation of these native varieties is increasing due to local and national government policies for the sustainable use of biodiversity and in response to the industrial exploitation of this fruit to obtain pulp and juice. This results in the generation of waste such as peels and seeds, which could be used to obtain products of interest in pharmacological, cosmetic, or food industries, promoting a circular economy [10,11,12,13,14,15].

Chilto fruits (pulp, seed, and peel) have a low sugar concentration and contain high amounts of fiber, vitamin C, carotenoids, and pigments such as anthocyanins [11]. Excellent health-promoting activities of ethanolic extracts obtained from chilto seed, pulp, and peel powders have been demonstrated, displaying antioxidant, anti-inflammatory, hypoglycemic, and hypolipemic activities attributed to phenolic acids such as rosmarinic and caffeoylquinic acid derivatives [10,11,12,16,17,18]. Previous reports studied the composition and functionality of pectic polysaccharides and hydrocolloids from orange chilto mucilage and pulp [19,20,21]. Recently, red chilto pectin was extracted from the peel and partially characterized. The pectin-enriched extract presented excellent film-forming properties that could be used in the food industry [17]. For this, the present study aimed to obtain and physicochemically and functionally characterize the pectin from the red chilto peel, a waste product obtained in the industrialization of red chilto.

## 2. Results and Discussion

**Figure 1:**
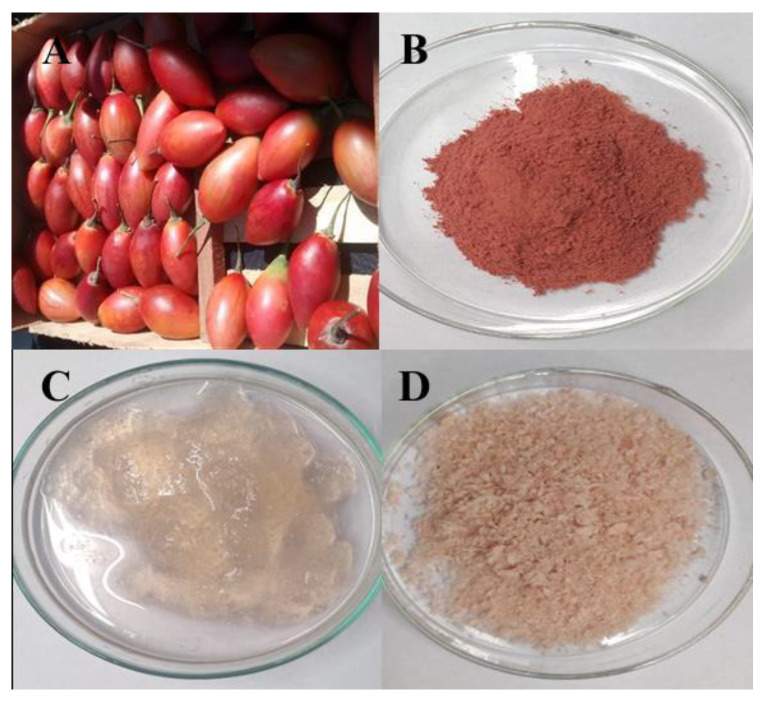
Red chilto fruits (**A**); peel powder of red chilto fruits (**B**); macroscopic image of chilto peel hydrocolloid (**C**); macroscopic image of freeze-dried pectin (**D**).

Nowadays, tons of fruit and vegetable waste is generated containing considerable amounts of valuable components that can be harnessed to benefit the food, pharmaceutical, and cosmetic industry and reduce environmental concerns such as global warming. Numerous agricultural by-products and wastes contain economically valuable pectin [22]. This work was focused on the study of the extraction and characterization of the pectin fraction of red chilto peel, a by-product of the food industry of Northwestern Argentina and South America. Figure 1 shows the general appearance of the fresh chilto peel extracted pectin before and after the freeze-drying process.

### 2.1. Red Chilto Pectin Morphology, Viscosity, and Color Parameters

**Figure 2:**
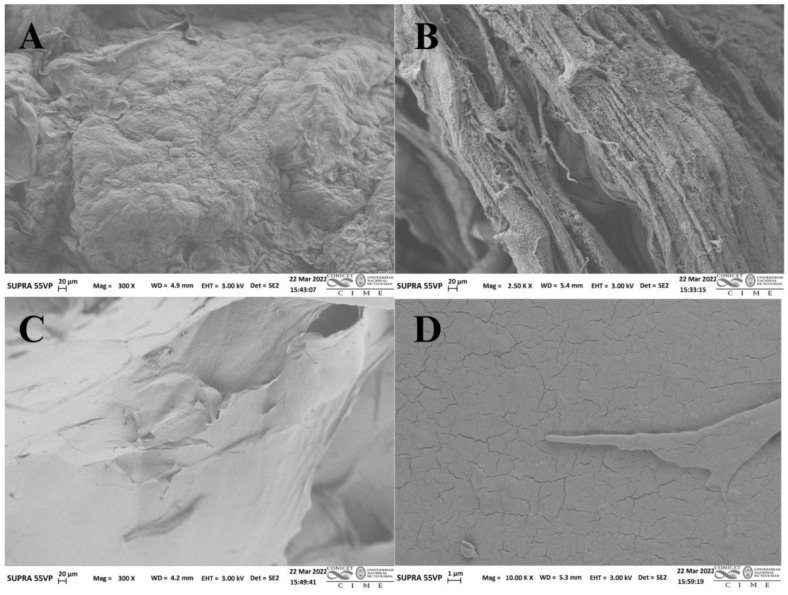
Scanning electron micrograph of red chilto wet pectin surface (**A**), wet pectin fibrous region (**B**), and red chilto dry pectin (**C**,**D**).

The color of the pectin is considered a crucial parameter because it contributes to the appearance of the obtained solution or gel and, therefore, the final appearance of the pectin-added food products and bioformulations [23]. The color parameters are shown in Table 1. The extracted pectin had a lighter appearance (high values of L*, 75.4). Regarding the a* values, positive scores indicate that the color of the pectin extract shifted toward reddish. Some pectins may contain compounds, such as polyphenols or water-soluble pigments, trapped in their structure during extraction, which confer color to pectins [23]. The extracted wet pectin (Figure 2A) has a smooth and compact nanostructure with small round shapes. It presents a fibrous region (Figure 2B), which indicates that the extracted pectin is heterogeneous. The pectin dried via lyophilization verified that the drying has some effects on its structure and angular mounds, and a cracked surface can be observed (Figure 2C,D). Also, after lyophilization, the extracted pectin became porous and presented some microfractures. Liew et al. [24] showed similar results for wet and dry pectin from passion fruit peel. Hydrocolloids from orange chilto puree extracted with 72% ethanol presented a surface morphology similar to pectin from red chilto peel [20]. In any case, the pectin drying process helps its storage for later use, which may benefit the food and cosmetic industry.

### 2.2. Chemical Composition of Red Chilto Pectin

The pectin recovery yield from red chilto peel was around 24%, which was higher than that reported for the same fraction of hydrocolloid extracted from orange chilto pulp (9%) and orange chilto puree with a different extraction method (8.35%) [25,26]. Table 1 summarizes the chemical characterization of the red chilto pectin extract. Many of the properties of the pectin could be affected by the quantity of total phenolic compounds co-extracted with this biopolymer, such as surface tension, emulsifying capacity, and biological activities [27]. The value of TPC co-extracted with pectins was 40.0 mg GAE/100 g DW pectin. This value was similar to that reported for pectin from orange peel (39.95 mg/g pectin) [28]. The content of co-extracted phenolic compounds represents only 2.3% of the total phenolic compounds extracted from 100 g of chilto peel (408.9 ± 2.3 mg/100 g of peel powder) [12]. The content of anthocyanins co-extracted with red chilto pectin was approximately five times lower than the content of TPC (Table 1). Pectin polysaccharides interact with anthocyanins via hydrophobic forces and hydrogen bonds. The latter are formed between hydroxyl groups of anthocyanins and non-esterified galacturonic acid in the pectin structure. In addition, the stability of anthocyanins is significantly improved by interacting with pectin molecules [29]. There are reports that the predominant anthocyanin in red chilto peel (cyanidin-3-glucoside) can bind to blueberry pectin and improve its stability during the digestive process [30,31].

The total sugar content in the pectin was 22.01% (Table 1). This carbohydrate content was similar to that reported for pectin extracted from *Averrhoa bilimbi* (25.7%), sour cherry pomace (26.4%), and tomato (*Solanum lycopersicum*) peel (19.1–23.5%), while other principal sources of pectin such as citrus peel had a higher content of total sugars (71–74%) [32,33,34,35]. Evidence suggests that the quality and quantity of these compounds depend on the pectin source and extraction method due to the broad variability reported by different researchers. The protein and ash in red chilto pectin were 8.1 and 4.7 g/100 g pectin, respectively (Table 1). These values were higher than banana peel and pistachio pectin [36,37]. The Food and Agriculture Organization indicates that the protein content in pectin should not exceed the value of 15.6 g/100 g, and in addition, the proteins can affect the emulsifying properties of pectins [38]. Regarding the monosaccharide composition, chilto pectin contained typical pectin components, mainly galacturonic acid, like arabinose, rhamnose, and galactose (Table 1). A similar chemical composition was found in pectin from eggplant peel [6]. Since galactose and arabinose were dominant among the neutral sugars of red chilto pectin, it can be concluded that galactans and arabinans are the prevailing side chains in the pectin structure obtained from the chilto by-product. Similar results were reported in the pectin fraction of orange chilto pulp [21]. Red chilto pectin showed a high degree of methoxylation (MD) of 60.8%. This value conditions rheological behavior, gelling properties, and interfacial activities displayed by pectins. In sucrose and low pH values, highly methoxylated pectin (HMP) can form gels, a property appreciated in various gelling foods such as jams and jellies [39]. The pectins obtained from chilto by-products could be used as a provider of structure in pastes, ointments, oils, and creams in the cosmetic industry. In addition, the pectin extract could be an attractive ingredient source for the elaboration of cosmetics as a thickener and stabilizer for formulations such as body lotions, shampoos, and conditioners.

Table 1 shows the results of the apparent viscosity of aqueous solutions of red chilto peel pectin at concentrations of 0.5, 1.5, and 3% (*w*/*w*). When large polysaccharide molecules are hydrated in aqueous solutions, they occupy large volumes, affecting the viscosity of these solutions. It is expected that pectin solutions are presented as non-Newtonian pseudoplastic fluids, showing a shear-thinning behavior characterized by decreasing the viscosity of the pectin solution with an increasing shearing rate [40]. As was predictable, the viscosity of chilto pectin in aqueous solutions increases with concentration. The increment in the viscosity observed with the increase in pectin concentration could be related to the increased availability in hydroxyl groups and, therefore, a higher number of formed hydrogen bonds that conduce to a more entangled net of polymer molecules limiting their movement [41].

### 2.3. Functional Properties of Red Chilto Red

WHC and OHC represent the capacity of a material to retain water and oil; the values for red chilto pectin are shown in Table 2. The WHC of chilto pectin was 4.19%, much higher than apple pomace pectin and almond by-products [42,43]. A high value of WHC indicates that pectin retains more water and could help improve texture properties, increase bulk volume, and avoid syneresis problems in some food systems. OHC is a relevant parameter in the characterization of pectins since a high value of OHC facilitates the dispersion of immiscible liquids indicating that pectin can be used as a stabilizer and emulsifier in fatty food products [44]. The OHC measurement of red chilto pectin was similar to that of *Opuntia ficus indica* and commercial citrus pectin (1.23 and 0.93%, respectively) [45] (Table 2).

To further evaluate the functionality of red chilto pectin, the emulsifying capacity (EC) and emulsion stability (ES) were measured (Table 2). The EC of the pectin was similar to that of orange chilto hydrocolloid (84.7%) and much higher than citrus pectin, carrageenan, and walnut by-products [25,46]. A biopolymer is considered a proper emulsifying agent when its EC is higher than 50%. Furthermore, previous studies indicate that the protein content and the MD of pectin have a positive effect on EC [47]. Some reports showed that the emulsifying capacity of pectins is highly related to the presence of residual hydrophobic proteinaceous components in the pectin structure [48]. In the case of ES, the red chilto pectin emulsion was highly stable at room temperature (Table 2), and therefore, it would be possible to apply pectin as an excellent emulsifying agent in the food industry, comparable with other conventional sources of pectin. The EC and ES of pectin-enriched extract (PEE) also indicates the pectin’s potential to bind to bile acids, one of the principal mechanisms for reducing blood cholesterol levels [49].

The foam is formed by trapping air bubbles in the liquid, semi-solid film, or solid substance, so it can be defined as a spatially homogeneous dispersion. Foaming properties (capacity and stability) are fundamental for aerated food materials, such as ice cream, with a high degree of swelling [50]. The FC and FS of PEE were 21.10 and 79.06%, respectively. The FC value was higher than the sunflower by-product pectin [51]. It is necessary to highlight the high value of FS, which successfully maintains the foam volume after 30 min and could be used in foam-based food products such as mousses, milkshakes, ice cream, and marshmallows. Kazemi et al. [37] mentioned that the high foaming capacity and stability could be due to high concentrations of phenolic compounds and the drop in pectin surface tension levels.

### 2.4. Antioxidant Activity of Chilto Pectin

Red chilto pectin can display antioxidant activity by the scavenging of ABTS^•+^ or O_2_^•−^ radicals, depuration of H_2_O_2_, or the inhibition of xanthine oxidase (Table 3). The chilto pectin showed a considerable ABTS^•+^ scavenging ability exhibiting an SC_50_ value of 0.51 mg/mL. The red chilto pectin showed greater ABTS^•+^ scavenging capacity than that reported for sunflower by-products (SC_50_ = 2.88 mg/mL) or citrus pectins (SC_50_ > 3 mg/mL) [51,52]. There are reports that the hydroxyl groups of the galacturonic acid units can donate protons to reduce a radical, which could explain the antioxidant activity of pectins, which increase with the increment in galacturonic acid content [53,54]. Reactive oxygen species (ROS) such as superoxide anion (O_2_^•−^) or H_2_O_2_ can react with any biomolecules and cause considerable damage because of their high activity. The antioxidant activity of chilto pectin against O_2_^•−^ and H_2_O_2_ showed dose–response behavior, with an SC_50_ value of 5.2 and 5.6 mg/mL, respectively, similar to the report of Xiong et al. [55] for acid-soluble pectin from okra (*Abelmoschus esculentus*). In addition, the presence of phenolic compounds co-extracted with red chilto pectin, known as natural antioxidants, can increase the antioxidant activity of pectin.

### 2.5. Hypoglycemic Activities of Chilto Pectin

Nowadays, natural compounds that inhibit α-amylase and α-glucosidase enzymes have attracted much attention [56]. These enzymes are responsible for the degradation of dietary carbohydrates into simple monosaccharides and hence are one of the first targets for delaying glucose absorption and decreasing glycemia levels [57]. The pectin inhibited the α-amylase enzyme with an IC_50_ value of 3.3 mg/mL, similar to that reported for pectin from *Opuntia macrorhiza*, but not α-glucosidase [58] (Table 3). In previous works, the inhibitory capacity of high-methoxylated pectins on digestive enzymes was reported and compared with low-methoxylated pectins, showing mechanisms that involve superficial and non-competitive hydrophobic interactions [59]. On the other hand, the monosaccharide composition of pectin would also be related to the inhibitory capacity of α-amylase, particularly the glucose contents in the pectin structure [57]. A diet rich in pectin increases the viscosity of the ingested food, thus slowing the diffusion of amylase and hydrolysis products and hindering amylase access to starch substrates.

**Figure 3:**
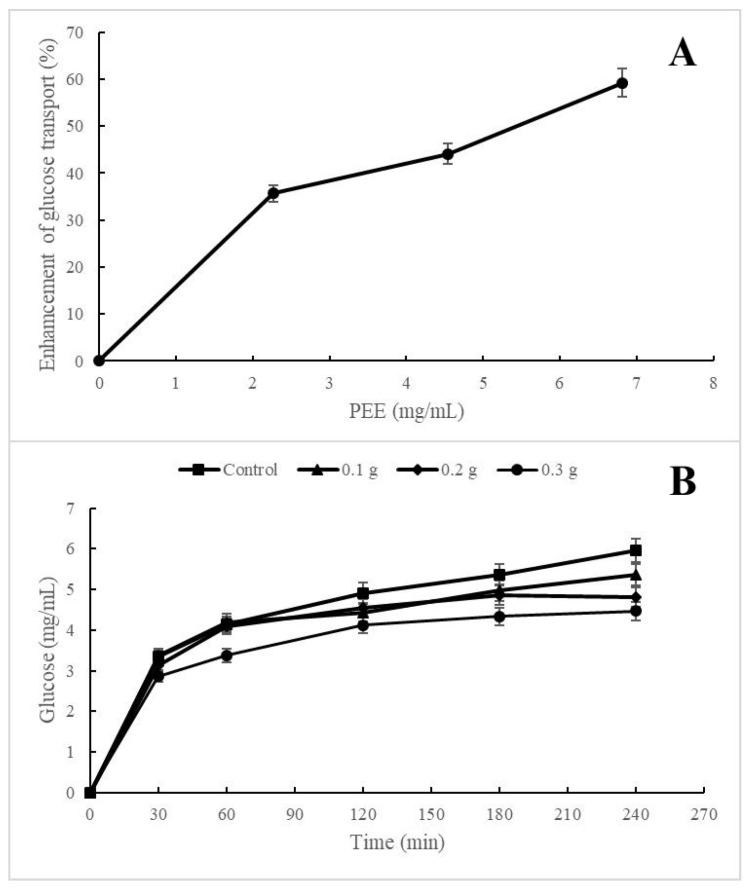
Hypoglycemic activity of PEE extract. Effect of PEE on glucose uptake by *S. cerevisiae* (**A**). Effect of different amounts of pectin on glucose diffusion (**B**). Values are reported as mean ± standard deviation of triplicates.

Pectic polysaccharides are characterized by resisting the hydrolytic action of enzymes and not being altered during their passage through the gastrointestinal tract [60]. The effect of the pectin concentration on cellular glucose uptake is shown in Figure 3A. In the graph, dose–response behavior can be observed, reaching an improvement of 60% in the cellular uptake of glucose at a concentration of 6.8 mg/mL of pectin. This effect may be due to an interaction mechanism between pectin and glucose transporters in the *Saccharomyces* cell. It was extensively reported that pectins improve insulin sensitivity and lower blood glucose levels [60,61]. In addition, some studies demonstrate the potential of citrus pectin to decrease blood glucose levels and insulin resistance in diabetic rats after four weeks of administration and suggest that the mechanism could be regulated by the expression of the PI3K/Akt signaling pathway [60]. Researchers reported that 20 g of apple pectin per day improves glucose tolerance in patients with type 2 diabetes [62].

Figure 3B shows the ability of the pectins to retard the diffusion of glucose through the membrane with higher activity as the pectin concentration increases. When pectins pass through the gastrointestinal tract, they form a complex three-dimensional matrix with fibrous and amorphous characteristics, the structure of which will depend on the physicochemical properties, such as the degree of methylation, the degree of acetylation, the distribution of molecular weight, the distribution of unmethylated galacturonic acid residues, and gel-forming ability [63]. However, complementary assays are necessary to determine the hypoglycemic activity of red chilto pectin.

### 2.6. Toxicity Assessment

Pectin is recognized as safe for human consumption by the Codex Alimentarius standard from FAO-WHO, with no recommended maximum pectin daily intake specified. Also, in the USA, pectin is classified as GRAS (Generally Recognized As Safe) by the FDA, carrying the highest level of approval possible under the GRAS program, which means pectin can be used freely in food and beverage products. Recently, chronic and subchronic toxicity studies on animals revealed that the highest doses showing no adverse effects were between 12 and 16 times the recommended intake of dietary fiber for humans by EFSA [64,65]. However, despite these recognitions of pectin being safe, this work evaluated the toxicity of the red chilto pectin in popular organism models, like *Artemia salina* and *Caenoharbitis elegans* models, the results of which correlated with toxicity data on animals and humans but also provided information concerning pathways of toxicity and modes of toxic action [66,67]. *A. salina* is a marine zooplanktonic crustacean used to test the acute toxicity of bioactive molecules, natural extracts, nanoparticles, and several toxic materials. The nematode *C. elegans* represents a compelling alternative model to predict compound toxicity using a whole animal with conserved processes with mammals, which has been used for years to carry out studies on genetics, developmental biology, or neuronal activity [66]. In both acute toxicity assays, none of the extracts at the concentrations tested caused mortality concerning the negative control (distilled water or M9 buffer). With the highest concentration tested (50 mg/mL, 5%), the viability of *A. salina* and *C. elegans* was practically unaffected. Ferreira-Gonçalves [68] evaluated the toxicity of different concentrations of apple pectin and observed that those above 3% (wt.) had high mortality rates on *A. salina*. Other reports indicated that high concentrations of citrus peel pectin were not toxic to *A. salina* organisms [69]. Considering the WHO classification for hazardous compounds, pectin extracts can be classified as “practically non-toxic” [70]. It should be noted that live nauplii larvae did not change their locomotor behavior during incubation. The evaluation of pectin obtained from *S. betaceum* residue indicates it is safe to use, which provides added worth beyond its functional value to this biopolymer.

## 3. Materials and Methods

### 3.1. Fruits Samples

*S. betaceum* fruits (red variety) with the same degree of ripeness were collected at Finca del Obispo (Villa Jardín de Reyes, Jujuy, Argentina) during the month of December 2018 (Figure 1A). The specimens were included in the Phanerogamic Herbarium of the Miguel Lillo Foundation (LIL-HbF), Tucumán, Argentina (Voucher number 617.907/LIL, 24°10′1.5″ S 65°23′40.3″ W). The fruits were considered ripe when they had uniformity in the peel color throughout the fruit and a smooth texture to the touch, features that fruits usually have when they are consumed. The three parts of the fruit (peel, pulp, and seed) were separated and freeze-dried. The peel powders (Figure 1B) obtained were vacuum-packed and stored at −20 °C.

### 3.2. Extraction of Red Chilto Pectin

The pectin was obtained from the red chilto peel powder according to the methodology described by Orqueda et al. [17]. Briefly, 10 g of red chilto peel powder was dispersed in 300 mL of hot distilled water for 2 h at 100 °C. Then, the mixture was centrifuged at 1500× *g* for 20 min. The supernatants were combined, and the polysaccharides were precipitated with absolute ethanol (2 vol) and recovered by centrifugation at 3000× *g* for 10 min (Figure 1C). The resulting polysaccharide was freeze-dried (pectin-enriched extract, PEE) (Figure 1D), and the recovery yield was calculated.

### 3.3. Physicochemical Characterization of Red Chilto Pectin

#### 3.3.1. Sugar, Proteins, Anthocyanins, and Total Phenolic Compounds Quantification

Total sugars were determined using the phenol-sulfuric acid method [11]. Protein analysis was performed using the Kjeldahl method [71]. Anthocyanins content was determined according to the methodology described by Costamagna et al. [72]. Total phenolic compound (TPC) content was determined using the Folin–Ciocalteau reagent [73].

#### 3.3.2. Color, Ash, and Moisture Determination

Chromatic parameters were measured with a Chroma meter CR-400 (Konica Minolta, Tokyo, Japan) colorimeter using the CIELab system. Results were expressed as chromaticity coordinates L*, a*, and b* (objective parameter). The L* coordinate represents the luminosity of the sample (it presents values between 0 and 100); the parameter a* represents the contribution of green or red, and b* represents the contribution of blue or yellow. The ash content was determined via a gravimetric assay [74]. The moisture content in the dried powders was determined via oven drying at 105 °C until a constant weight was reached, according to the AOAC method [75].

#### 3.3.3. Apparent Viscosity Measurement

The apparent viscosity (η_ap_) of red chilto pectin solutions was measured at different concentrations. Solution viscosity was measured using a viscometer (Brookfield DVII + ProEXTRA, Brookfield Engineering Laboratories, INC., Middleboro, MA, USA). Briefly, 5 mL of each concentration of aqueous pectin solution (0.5, 1.5, and 3%) was placed into the stainless steel cup of the viscometer, and then the apparent viscosity value was obtained using a S18 spindle at 25 °C. Spindle selection was adjusted to be within 10–90% torque value. The results were expressed in mPa.s.

### 3.4. Morphology via Scanning Electron Microscopy

Scanning electron microscopy (SEM) was performed using a ZEISS SUPRA-55 VP field-emission scanning electron microscope at Centro Integral de Microscopía Electrónica (CIME), CONICET-UNT. The samples were vacuum sprayed with a mixture of gold and palladium to allow conductivity and were observed under SEM. The surface microstructure of chilto pectin was studied at 3 kV accelerating voltage and using 300×, 2.5, and 10 Kx magnification.

### 3.5. Functional Properties of Chilto Pectin

#### 3.5.1. Water and Oil Holding Capacity

The water and oil holding capacities (WHC and OHC) of the chilto pectin were determined following the protocol described by Hosseini, Khodaiyan, Kazemi, and Najari [28]. Briefly, 0.15 g of sample was weighed, and 15 mL of water or commercial sunflower oil was added, stirred, and left at room temperature for 1 h. The mixture was centrifuged at 3000× *g*, the supernatant removed, and the residue dried on filter paper and weighed. WHC was expressed as g of water retained per g of sample, while OHC was expressed as g of oil held per g of sample.

#### 3.5.2. Emulsifying Capacity and Stability

The emulsifying capacity (EC) of chilto pectin was measured by Bayar et al. [45]. In brief, 5 mL of 2% pectin was mixed with 5 mL of sunflower oil. The mixture was blended vigorously in a vortex for 1 min and centrifuged at 4000× *g* for 5 min. Then, the volume of the obtained emulsion was measured. The emulsifying stability (ES) was measured by heating emulsions at 80 °C for 30 min in a water bath, then cooling to room temperature, and centrifuging at 1200× *g* for 5 min. EC and ES were calculated using the following equations:

EC = (Volume of the emulsion/total volume (mL)) × 100

ES = (Volume of remaining emulsion/volume of original emulsion (mL)) × 100

#### 3.5.3. Foaming Properties

The foaming properties of pectin were measured in accordance with the protocol described by Kazemi, Khodaiyan, and Hosseini [6]. In summary, 10 mL of pectin solution (4%) was placed in polypropylene centrifuge tubes and vortexed for 3 min. Foaming capacity (FC) and foam stability (FE) were calculated according to the following equations:

CF (%) = (V_0_ − V_T_/V_T_) × 100

EF (%) = (V_30_ − V_T_/V_T_) × 100

V_0_: Volume after vortex
V_30_: Volume after 30 min
V_T_: Total volume of reaction

### 3.6. Antioxidant Activity

#### 3.6.1. Total Antioxidant Activity of Red Chilto Pectin

The total antioxidant activity of pectin was evaluated with the ABTS (2,2-azinobis (3-4 ethylbenzothiazoline)-6-sulfonic acid) radical-cation (ABTS^•+^) method [11] using different concentrations of PEE (0.1–1 mg/mL). The results were expressed as the concentration of pectin necessary to scavenge 50% of ABTS cation radical (SC_50_).

#### 3.6.2. H_2_O_2_ Scavenging Assay

The hydrogen peroxide scavenging activity of pectin was measured spectrophotometrically at 504 nm [12]. Different concentrations of pectin (1–10 mg/mL) and H_2_O_2_ were pre-incubated for 3 min at 37 °C. Afterward, phenol solution (12 mM) and 4-aminoantipyrine was added to the reaction mixture. The content of hydrogen peroxide formed was determined at 504 nm. The results were expressed as the concentration of pectin necessary to scavenge 50% of H_2_O_2_ (SC_50_).

#### 3.6.3. Superoxide Radical Scavenging Assay

The capacity of pectin (1–10 mg/mL) to scavenge superoxide radicals was determined according to Cardozo et al. [76] by using the NADH/phenazine methosulfate (PMS)/nitro blue tetrazolium chloride (NBT) system and different concentrations of PEC extract. The reduction in NBT by superoxide radicals was measured spectrophotometrically at 560 nm.

#### 3.6.4. Xanthine Oxidase Inhibition

The inhibitory activity of pectin (0.50–5 mg/mL) on xanthine oxidase (XOD) activity was evaluated following the protocol described by Zampini et al. [77]. The results were expressed as the concentration of pectin necessary to produce a 50% inhibition of XOD activity (IC_50_).

### 3.7. Antihyperglycemic Activity

#### 3.7.1. Inhibitory Effect on α-Glucosidase and α-Amylase Activities

Enzyme inhibition assays were performed following the protocol described by Costamagna et al. [78]. The α-glucosidase enzyme was pre-incubated with the pectin (0.5–5 mg/mL) for 10 min at 4 °C. The addition of the substrate, *p*-nitrophenyl α-D-glucopyranoside, started the reaction, and the mixture was incubated for 15 min at 37 °C. The absorbance was read at 405 nm in a microplate spectrophotometer. The α-amylase enzyme inhibition assay was performed using the Amilokit^®^ (Wiener Lab Group, Rosario, Argentina, Kit No. 1504163370). Before starting the reaction, the amylase was pre-incubated with different concentrations of pectin for 5 min at 4 °C. The absorbance was recorded at 640 nm in a spectrophotometer. The results were expressed as the concentration of pectin necessary to produce 50% inhibition of *α*-amylase activity (IC_50_).

#### 3.7.2. Activity on Glucose Diffusion and Glucose Intake by Saccharomyces Cerevisiae Cells

The effect on glucose diffusion of chilto pectin was measured according to Ahmed et al. [79]. Different pectin samples (0.25, 0.5, and 1 g) were mixed with 5 mL of 20 mM glucose. Then, each mixture was dialyzed against 40 mL of distilled water at 37 °C under stirring. The glucose content in the dialysate was measured at 30, 60, 120, 180, and 240 min using the enzymatic blood glucose kit (Wiener lab. 1400101). Negative control was performed without the addition of pectin.

The effect of red chilto pectin on the intake of glucose by *S. cerevisiae* cells was performed according to Bhutkar et al. [80]. First, yeasts were washed, and a suspension (10% *v*/*v*) was prepared in distilled water. The reaction consisted of 100 μL of yeast suspension prepared in contact with increasing concentrations of pectin extract. Then, 1 mL of 20 mM glucose solution was added to the mixture, incubated for 1 h at 37 °C, and centrifuged at 2500× *g* for 5 min. The glucose concentration in the supernatant was determined using an enzymatic glycemia kit (Wiener lab. 1400101). The percentage of increase in glucose consumption by *S. cerevisiae* cells was calculated using the following formula:

Increase in glucose uptake: (DO control − DO sample)/(DO control) × 100

### 3.8. Toxicity Tests

#### 3.8.1. Acute Toxicity Using Artemia Salina Test

The *A. salina* toxicity test has been used as a rapid detection assay on a laboratory scale. This method has proven to be highly advantageous due to its simplicity and low cost, its good correlation with other animal testing methods, and the possibility of evaluating a large number of samples at the same time and in a short period. Increasing concentrations of pectin extract (12.5–50 mg/mL) were used to evaluate its acute toxic effect utilizing the *A. salina* microplate assay [81]. Negative controls with distilled water and positive controls with potassium dichromate (10–40 µg/mL) were assayed. After 24 h of exposition, the number of dead shrimp in each well was counted.

#### 3.8.2. *Caenorhabditis elegans* Toxicity Assay

Nematode strains (*Caenorhabditis elegans*) N2 (Bristol) was obtained from the Genetics Center (University of Minnesota, Minneapolis, MN) and maintained at 20 °C on Nematode Growth Medium (NGM) supplemented with *Escherichia coli* strain OP50 (uracil requiring bacterial strain). To evaluate the acute toxicity of red chilto pectin on L1 larval-stage *C. elegans* nematodes, different concentrations of pectin extract (12.5–50 mg/mL) were tested in a 24-well plate, using M9 buffer solution (0.3% KH_2_PO_4_, 0.6% Na_2_HPO_4_, 0.5% NaCl, 0.012% MgSO_4_; pH 7.0) as a negative control. Briefly, 500 µL of M9 buffer, 100 µL of the different extract concentrations, and 10 µL of the nematode suspension containing approximately 60 individuals were placed in each well. The plates were incubated for 24 h at 18 °C. Worm viability was determined under a magnifying glass. Worms that did not show movement were marked as dead. Each assay was performed in triplicate.

## 4. Conclusions

In this study, red chilto fruit waste was exploited through the recovery of pectin, which showed suitable biological and functional properties. This pectin showed proper technological functionality displaying water and oil holding capacities and emulsifying and foaming capacities. This pectin contains biomolecules such as phenolic compounds, anthocyanins, and proteins, which could be responsible for its biological activities. This polysaccharide showed antioxidant activity against ABTS radical, superoxide anion, and H_2_O_2_, xanthine oxidase inhibition, and in vitro hypoglycemic potential (α-amylase enzyme inhibition). The biological, functional, and toxicological properties exhibited by red chilto pectin make it valuable for the suitable development of ingredients for functional foods, or health products, promoting a circular economy in Argentina around this fruit. However, further studies relating structure with chilto pectins’ function and the effect of several factors such as pH, temperature, and divalent ion concentration on the rheological properties of chilto pectin solutions are needed to design pectin formulations for smart bioapplications in pharmaceutical, cosmetic, or food industries.

## Figures and Tables

**Table 1:** Proximate composition of red chilto peel pectin.

**Chemical Composition**	
TPC ^1^ (%)	0.0400 ± 0.0020
Anthocyanin ^2^ (%)	0.0065 ± 0.0005
Ash (%)	12.2 ± 0.9
Protein (%)	4.7 ± 0.2
Total sugars (%)	22.1 ± 0.5
**Color parameters**	
L*	75.4 ± 0.33
a*	7.8 ± 0.06
b*	13.8 ± 0.1
**Apparent viscosity ^3^**	
0.5%	446.0 ± 3.0
1.5%	507.1 ± 2.8
3%	676.9 ± 2.1

^1^ TPC: Total phenolic compounds; g gallic acid equivalent/100 g pectin; ^2^ Anthocyanin: g cyanidin 3 glucoside equivalent/100 g pectin. ^3^ mPa.s of pectin solutions at different concentrations. Percentage: g/100 g pectin. Values are reported as mean ± standard deviation of triplicates.

**Table 2:** Functional properties of red chilto peel pectin.

**Functional Properties**	
WHC (g W/g DW)	4.19 ± 0.04
OHC (g O/g DW)	2.02 ± 0.09
EC (%)	83.00 ± 0.50
ES (%)	87.50 ± 0.62
FC (%)	21.10 ± 0.90
FS (%)	79.06 ± 1.20

W: water; O: oil; DW: dry weight; WHC: water holding capacity; OHC: oil holding capacity; EC: emulsifying capacity; FC: foaming capacity; FE: foam stability. Values are reported as mean ± standard deviation of triplicates.

**Table 3:** Antioxidant and hypoglycemic activities of red chilto peel pectin.

Antioxidant and Hypoglycemic Activities	SC_50_ or IC_50_ (mg/mL) ^1^
O_2_^•−^	5.20 ± 0.90
ABTS^•+^	0.51 ± 0.03
H_2_O_2_	5.64 ± 0.05
Xanthine oxidase	3.15 ± 0.08
α-glucosidase	NI ^2^
α-amylase	3.30 ± 0.10

^1^ SC_50_: concentration of PEE necessary to scavenge 50% of radicals (for O_2_^•−^, ABTS^•+^, H_2_O_2_); IC_50_: concentration of pectin necessary to inhibit 50% of enzyme activity (for xanthine oxidase, α-glucosidase, and α-amylase). ^2^ NI: not inhibited. Values are reported as mean ± standard deviation of triplicates.

## Data Availability

The datasets generated and/or analyzed during the current study are available from the corresponding author on reasonable request.
